# Immature wild orangutans acquire relevant ecological knowledge through sex-specific attentional biases during social learning

**DOI:** 10.1371/journal.pbio.3001173

**Published:** 2021-05-19

**Authors:** Beatrice Ehmann, Carel P. van Schaik, Alison M. Ashbury, Julia Mörchen, Helvi Musdarlia, Suci Utami Atmoko, Maria A. van Noordwijk, Caroline Schuppli

**Affiliations:** 1 Department of Anthropology, University of Zürich, Zürich, Switzerland; 2 Department for the Ecology of Animal Societies, Max Planck Institute of Animal Behavior, Konstanz, Germany; 3 Department of Biology, University of Konstanz, Konstanz, Germany; 4 Behavioral Ecology Research Group, Institute of Biology, Faculty of Life Science, Leipzig University, Leipzig, Germany; 5 Research Group Primate Behavioural Ecology, Department of Human Behavior, Ecology and Culture, Max Planck Institute for Evolutionary Anthropology, Leipzig, Germany; 6 Department of Biology, Graduate School, Universitas Nasional, Jakarta, Indonesia; 7 Faculty of Biology and Primate Research Center, Universitas Nasional, Jakarta, Indonesia; 8 Leipzig Research Center for Early Child Development, Leipzig University, Leipzig, Germany; 9 Development and Evolution of Cognition Research Group, Max Planck Institute of Animal Behavior, Konstanz, Germany; Emory University, UNITED STATES

## Abstract

As a part of growing up, immature orangutans must acquire vast repertoires of skills and knowledge, a process that takes several years of observational social learning and subsequent practice. Adult female and male orangutans show behavioral differences including sex-specific foraging patterns and male-biased dispersal. We investigated how these differing life trajectories affect social interest and emerging ecological knowledge in immatures. We analyzed 15 years of detailed observational data on social learning, associations, and diet repertoires of 50 immatures (16 females and 34 males), from 2 orangutan populations. Specific to the feeding context, we found sex differences in the development of social interest: Throughout the dependency period, immature females direct most of their social attention at their mothers, whereas immature males show an increasing attentional preference for individuals other than their mothers. When attending to non-mother individuals, males show a significant bias toward immigrant individuals and a trend for a bias toward adult males. In contrast, females preferentially attend to neighboring residents. Accordingly, by the end of the dependency period, immature females show a larger dietary overlap with their mothers than do immature males. These results suggest that immature orangutans show attentional biases through which they learn from individuals with the most relevant ecological knowledge. Diversifying their skills and knowledge likely helps males when they move to a new area. In sum, our findings underline the importance of fine-grained social inputs for the acquisition of ecological knowledge and skills in orangutans and likely in other apes as well.

## Abstract 2

To understand the development and evolution of cognition of our closest relatives, we need to investigate their learning behavior during every-day life. This study finds that wild orangutan males and females differ in their social learning strategies and subsequent learning outcomes from an early age, underlining the general importance of, and the effects of sex on, social learning in non-human great apes.

## Introduction

In many mammal species, immature individuals acquire subsistence skills through social learning [1–5]. Social learning appears to be particularly prominent in the foraging context, as immatures often closely observe competent individuals while feeding and solicit their food or tools [6–17]. With respect to who an immature observes, it has been suggested that among socially learning mammals, and specifically among primates, individuals go through 3 main phases of social learning (reviewed by [18]; see also [19,20–23]). During the first phase, basic subsistence skills are learned from key caregivers (in most cases, mothers). In the second phase, the pool of social learning targets expands to include other skilled group members or association partners. The third, nonobligatory, phase takes place after dispersal (i.e., the movement from birth to breeding site) when—upon encountering new conditions—immigrants may benefit from observing knowledgeable local residents. Data from a wide range of primates in the wild support the existence of the first 2 phases [6,8,11–14,24–27], while there is sparse, although very suggestive, evidence supporting the existence of the third phase [28–32]. However, whether an individual’s impending dispersal, or the absence thereof, also affects their attentional preferences and skill acquisition prior to the actual dispersal event, i.e., during the second phase of social learning, remains unexplored.

Irrespective of these 3 different phases, studies of several primate species have strongly suggested different sex-specific attentional biases (reviewed by [33]). In some species, immatures show a (most likely subconscious) preference to attend to adults of their own sex [34], whereas in others, immatures of either sex show social learning preferences for adults of one sex [35,36]. There are also species in which one sex seems to be generally more attentive and better at social learning (e.g., chimpanzees *(Pan troglodytes*) [37,38] and white faced capuchin monkeys (*Cebus capucinus*) [14]). In humans, children show a preference to attend to and learn from individuals of the same gender as soon as they can reliably differentiate between genders in others and themselves [39,40], and girls appear to be more attentive to social cues overall (e.g., [41] but see [42]).

Sex differences in social learning can be explained by several different mechanisms. Preferences to attend to the activities of same sexed adults may serve to learn sex-specific behaviors [34]. An overall increased attentiveness of one sex may be the result of differing physiological requirements, learning opportunities, and learning strategies [38,43,44]. In vervet monkeys, the preference of adult females as social learning targets over adult males was suggested to be linked to the fact that females are likely to possess the most ecological knowledge of the local area because, contrary to males, they spend their entire lives in their natal areas [35].

Sex-specific dispersal may indeed enhance sex-specific attentional biases in primates. Where sex-specific dispersal occurs, only one sex has to cope with the challenges of moving to, and settling in, a new physical and social environment [18,45,46]. Especially in species with long-distance dispersal, this new environment may differ from the natal area in many respects, including what, how, and where to eat [1,45,47]. Thus, attending to resident individuals in the new area is likely an important means by which individuals of the dispersing sex acquire local knowledge of that area [18,31]. However, sex-specific dispersal may also affect social interest before the dispersal event: It may be beneficial for members of the dispersing sex to learn from a large number of individuals so as to maximize the breadth of the knowledge and skill set they acquire, while it may be more beneficial for the philopatric sex to focus on acquiring local knowledge and skills.

Sex-specific attentional biases are expected to be most pronounced in species that show significant behavioral differences between sexes and also rely on large repertoires of socially learned skills. Dispersal is expected to have the greatest effect on learning in species that disperse over long distances. Orangutans meet all these criteria and are therefore an especially suitable species to look at sex-specific social learning and its potential outcomes. Orangutans show a pronounced sexual dimorphism with flanged males reaching twice the size of adult females, and they differ in several aspects of their activity budget (e.g., males travel more, have shorter active periods, and spend more time in each food patch and on the ground [48–50]). Most likely as a consequence of differing physiological requirements, adult female orangutans and flanged males differ in their macronutrient intake, caloric intake, and overall diet breadth [49,51,52]. Furthermore, male orangutans leave their natal areas before they start reproducing, and they are classified as long-distance dispersers [51,53]. After reaching sexual maturity, males spend several years in the unflanged phase, during which they roam between areas, before they transition into the flanged male phase [54]. Female orangutans, however, stay in their natal areas and settle into home ranges overlapping with their mothers’ and other maternal relatives’ [56–58].

Orangutans show vast repertoires of learned skills, in particular in the foraging context [57,58]. Orangutans take several years to acquire their foraging skills, and around the age of weaning, at 6.5 to 8.5 years, immatures spend as much time feeding as adults [60], and the full extent of their diet repertoires appear to have been established [17,61]. Overall, immature females acquire broader diets than their male peers which reflects their respective adult repertoire sizes [17,52]. The main mode of skill acquisition for immature orangutans is observational learning through peering (attentive and close range watching of a conspecific’s activities) followed by independent practice of the observed behavior [6,62]. Most peering happens during infancy (0 to approximately 8 years [6,63]). Young infants direct most peering at their mothers, and peering at individuals other than the mother increases with increasing age [6]. However, the details of the development of social attention directed at individuals other than the mother remain unclear.

The first aim of this study is to investigate whether immature female and male orangutans direct their attention to those individuals from whom they are most likely to acquire the ecological knowledge they will need in later life. Because adult male and female orangutans have different activity budgets and foraging behaviors, immatures are expected to show an attentional bias toward adults of their own sex. Furthermore, immigrant (i.e., nonresident) individuals might possess ecological skills and knowledge that will be relevant in the areas to which young males will eventually disperse. Therefore, a bias toward attending to unfamiliar individuals may allow immature males to acquire uniquely beneficial skills and knowledge, as well as facilitate later learning from unfamiliar individuals during and after dispersal. All in all, we expected that immature males are most attentive to the ecological skills and knowledge of individuals other than their mothers.

The second aim of this study is to examine how these potential differences in social attention are reflected in repertoires of acquired ecological skills and knowledge. We expected that because of their attentional focus on their mothers, immature females will show more similar repertoires to their mothers relative to immature males and their mothers. Furthermore, as a result of their increased social attention toward individuals other than their mothers, we expect immature males to acquire skills and knowledge outside of their mothers’ repertoires. The third aim of this study is to investigate whether immatures’ sex-specific attentional biases are limited to the feeding context or are rather more general biases toward attending to the activities of adults of their own sex.

To test these predictions, we investigated the development of social interest of immature orangutans in the Suaq and Tuanan populations (see Methods). We measured social interest in immatures at 2 levels: first, the allocation of peering events to different classes of individuals, and second, the share of association time that immatures spend in close proximity to different classes of individuals. Specifically, we predicted that in the feeding context, (i) immature males—relative to immature females—direct more peering at, and spend more time in close proximity to, individuals other than their mothers; and (ii) with respect to non-mother targets of social attention, immature males preferentially peer at adult males (i.e., unflanged males and flanged males) and immigrant individuals (including adult males and nonresident, independently ranging juveniles who are usually between 10 and 16 years old), whereas immature females preferentially peer at adult females and neighboring residents (i.e., adult females and resident independently ranging juveniles). In terms of the acquisition of dietary knowledge and skills, we predicted that (iii) by the end of their constant association with their mothers, immature females have a greater overlap with their mothers’ diets, whereas immature males eat a larger share of items that are not in their mothers’ diet repertoires. We further predicted that (iv) there are no sex differences in attention directed at activities outside of the feeding context (i.e., non-subsistence skills), suggesting that these attentional biases specifically serve to acquire relevant ecological knowledge.

## Results

### Immature males and females differ in the development of their social interest in non-mother individuals

**Fig 1:**
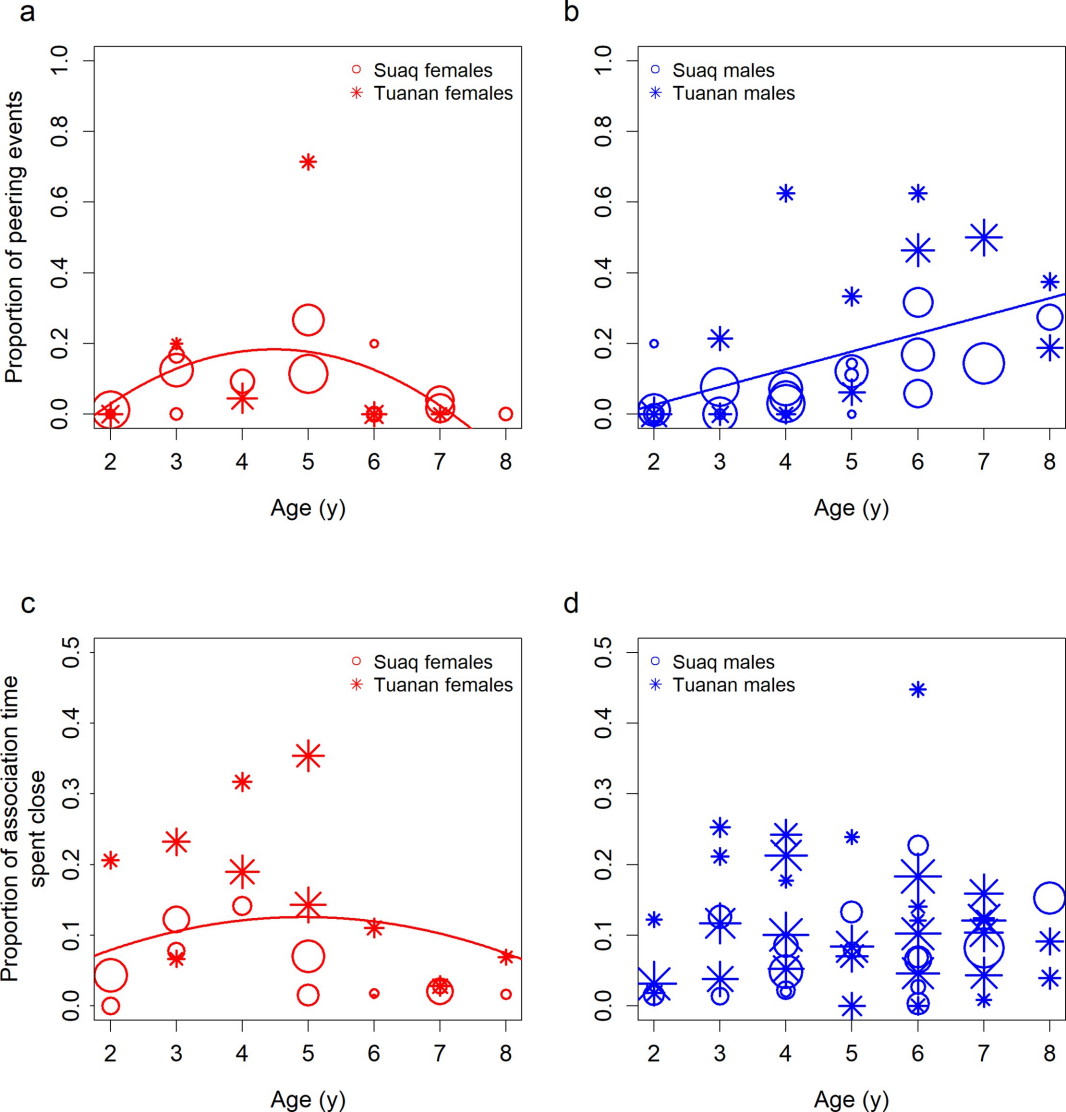
Development of social interest in immatures. Proportion of peering events in the feeding context that immature females (a) and males (b) directed at individuals other than their mother, over age, with *N* = 56 peering proportions based on 2,355 peering events by the immatures (802 events by immature females and 1,548 events by immature males). The proportion of association time that immature females (c) and males (d) spent in close proximity to individuals other than the mother, over age, with *N* = 71 association proportions based on 3,983 association hours (1,051 hours of immature females and 2,932 hours of immature males) of the immatures with non-mother association partners. The lines depict the best model fit for each sample. Symbol sizes correspond to sample sizes (i.e., number of peering events) for (a) and (b) and number of association hours for (c) and (d). The underlying data for this figure can be found in S1 Data.

When comparing overall peering rates of immature males and females in the feeding context, we found no differences between the sexes (S1 Fig, GLMM_Gaussian_: P_SexMale_ = 0.997, Estimate_SexMale_ = 0.0002, Std.Error_SexMale_ = 0.069; P_Age_ = 0.100, Estimate_Age_ = −0.467, Std.Error_Age_ = 0.072; P_SiteTuanan_ < 0.001, Estimate_SiteTuanan_ = −0.370, Std.Error_SiteTuanan_ = 0.073, *N* = 63 peering rates based on 1,094 peering events by the immatures). However, immature males and females differed in how they allocated their peering in the feeding context to different target individuals. During early infancy, both sexes directed most peering at their mothers, but over the first 3 years of life, the proportion of peering directed at individuals other than the mother gradually increased. Whereas among females, the proportion of peering directed at individuals other than the mother peaked during mid-infancy and then decreased, it steadily increased among males. For immature males, the best fitting model included a linear age term, and for the females, a quadratic age term (Fig 1A and 1B, Table 1).

**Table 1:** Model summaries.

Nr	Dependent variable	Sex	Effect	Effect type	Estimate	Std.Error	*P* value
(a)	Proportion of peering directed at non-mother individuals	Females	Intercept	Intercept	0.179	0.051	**<0.001**
			Age^2^	Fixed	−0.089	0.036	**0.014**
			Individual	Random	-	-	-
		Males	Intercept	Intercept	0.099	0.033	**<0.001**
			Age	Fixed	0.092	0.026	**<0.001**
			Site	Fixed	0.129	0.052	**0.012**
			Individual	Random	-	-	-
(b)	Proportion of association time spent in close proximity of non-mother individuals	Females	Intercept	Intercept	0.069	0.038	0.073
			Age^2^	Fixed	−0.017	0.008	**0.028**
			Site	Fixed	0.116	0.053	**0.028**
			Individual	Random	-	-	-
		Males	Intercept	Intercept	0.058	0.032	0.070
			Site	Fixed	0.080	0.040	**0.044**
			Individual	Random	-	-	-

Effects of age on (a) peering proportions directed at individuals other than the mother for immature females and males, with *N* = 56 peering proportions based on 2,350 peering events in the feeding context by the immatures (802 events by immature females and 1,548 events by immature males); (b) the proportion of association time immature females and males spent in close proximity (i.e., within 2 meters) of individuals other than the mother, with *N* = 71 association proportions based on 3,983 association hours of the immatures with non-mother association partners (1,051 hours of immature females and 2,932 hours of immature males); analyzed with GLMMs with a Gaussian family distribution. Significant *P* values at the 5% criterion are bolded.

GLMM, generalized linear mixed model.

As an additional measure of social interest, we looked at association patterns of immature males and females with non-mother association partners. We found no differences in overall association rates according to the immatures’ sexes (S2 Fig, GLMM_Gaussian_: P_SexMale_ = 0.360, Estimate_SexMale_ = 0.059, Std.Error_SexMale_ = 0.064; P_Age_ = 0.043, Estimate_Age_ = −0.061, Std.Error_Age_ = 0.030; P_SiteTuanan_ < 0.001, Estimate_SiteTuanan_ = −0.240, Std.Error_SiteTuanan_ = 0.064, *N* = 119 association rates based on 16,598 observation hours of the immatures). We also found no sex differences in rates of association with any of the different classes of non-mother association partners (S1 Table). However, the proportion of association time that immatures spent in close proximity (i.e., within 2 meters) of individuals other than their mothers partly reflected the sex difference we found for peering: While the proportion of association time spent in close proximity to others peaked during mid-infancy and then decreased among immature females, it remained constant among immature males. For immature females, the best fitting model included a quadratic age term, and for males, no age term (Fig 1C and 1D, Table 1).

### Immature males and females attend to different non-mother individuals

**Fig 2:**
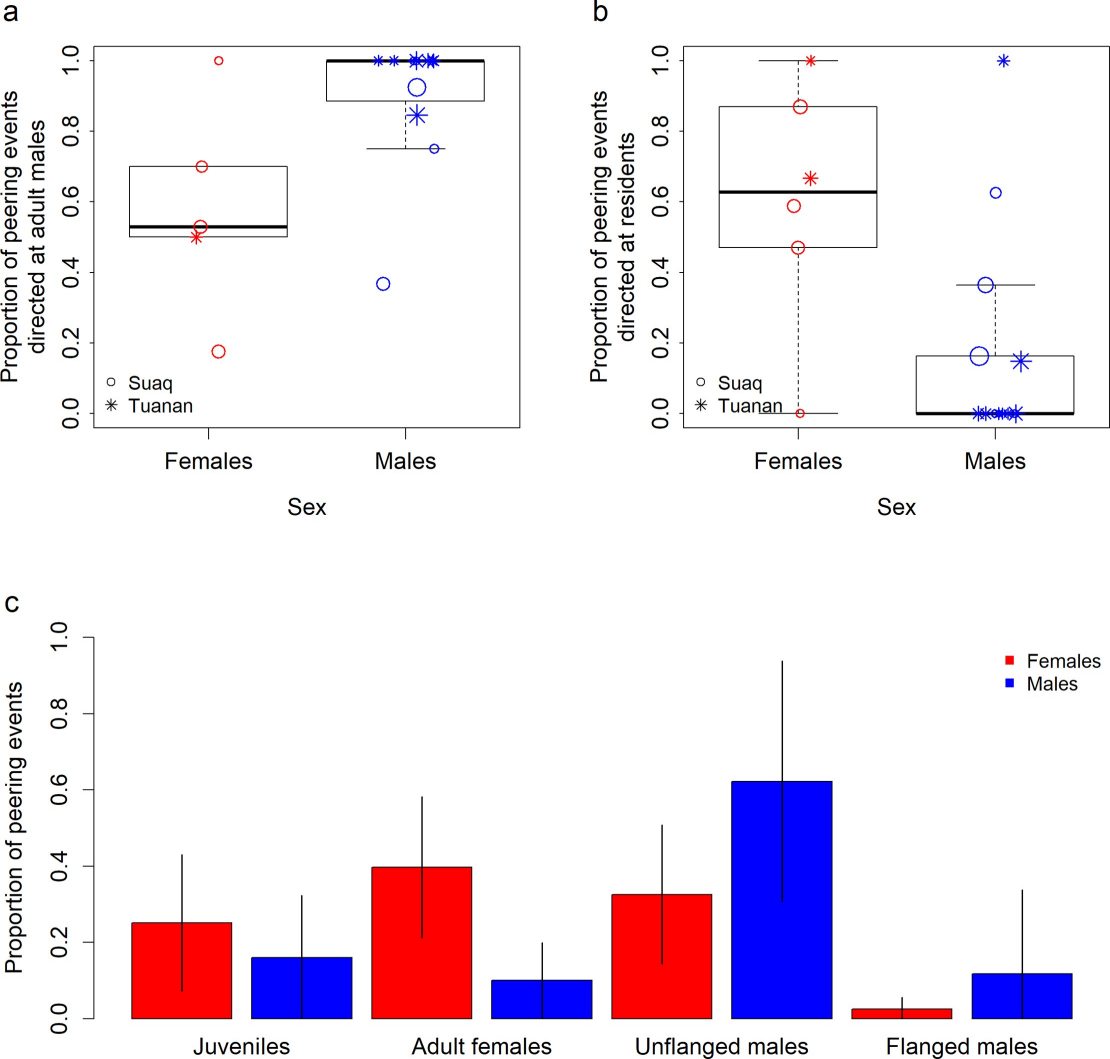
Immatures’ non-mother peering target choice. (a) Proportion of peering events that immature females and males at both sites directed at adult male versus female individuals other than their mothers, with *N* = 198 peering events (50 events by immature females and 148 events by immature males). (b) Proportion of peering events that immature females and males of both sites directed at resident versus immigrant individuals other than their mothers, with *N* = 249 peering events (66 events by immature females and 183 events by immature males). (c) Proportion of peering events that immature females and males of both sites directed at different age sex classes of non-mother individuals (excluding infants), based on *N* = 237 peering events directed at non-mother individuals (63 events by immature females and 174 events by immature males). Note that for (a) and (b), the analyses are based on binomial models and thus on peering events themselves and not on the proportions of peering events used here (see Results and Methods). For (a) and (b), symbol sizes correspond to the number of peering events per data point. The underlying data for this figure can be found in S1 Data.

With respect to non-mother adult peering targets, there was no significant difference between immature males and females in their odds of peering at adult males (GLMM_Binomial_: P_SexMale_ = 0.111, Estimate_SexMale_ = 1.439, Std.Error_SexMale_ = 0.846; P_SiteTuanan_ = 0.200, Estimate_SiteTuanan_ = 1.143, Std.Error_SiteTuanan_ = 0.893; P_Age_ = 0.049, Estimate_Age_ = 0.565, Std.Error_Age_ = 0.287, *N* = 198 peering events in the feeding context by the immatures at non-mother individuals; Fig 2A).

However, immature males had significantly higher odds of peering at immigrant individuals (including adult males and independent juveniles of both sexes) than immature females (GLMM_Binomial_: P_SexMale_ < 0.001, Estimate_SexMale_ = 2.205, Std.Error_SexMale_ = 0.651; P_SiteTuanan_ = 0.494, Estimate_SiteTuanan_ = 0.405, Std.Error_SiteTuanan_ = 0.591; P_Age_ = 0.689, Estimate_Age_ = −0.199, Std.Error_Age_ = 0.482; P_Sex:Age_ = 0.003, Estimate _Sex:Age_ = 1.590, Std.Error _Sex:Age_ = 0.539, *N* = 249 peering events in the feeding context by the immatures at non-mother individuals; Fig 2B).

Overall, immature females directed the highest proportion of their non-mother peering in the feeding context at resident adult females, and immature males directed the highest proportion of their non-mother peering at unflanged males (Fig 2C).

### At the end of dependency, immature females and males differ in their degree of dietary overlap with their mothers

**Fig 3:**
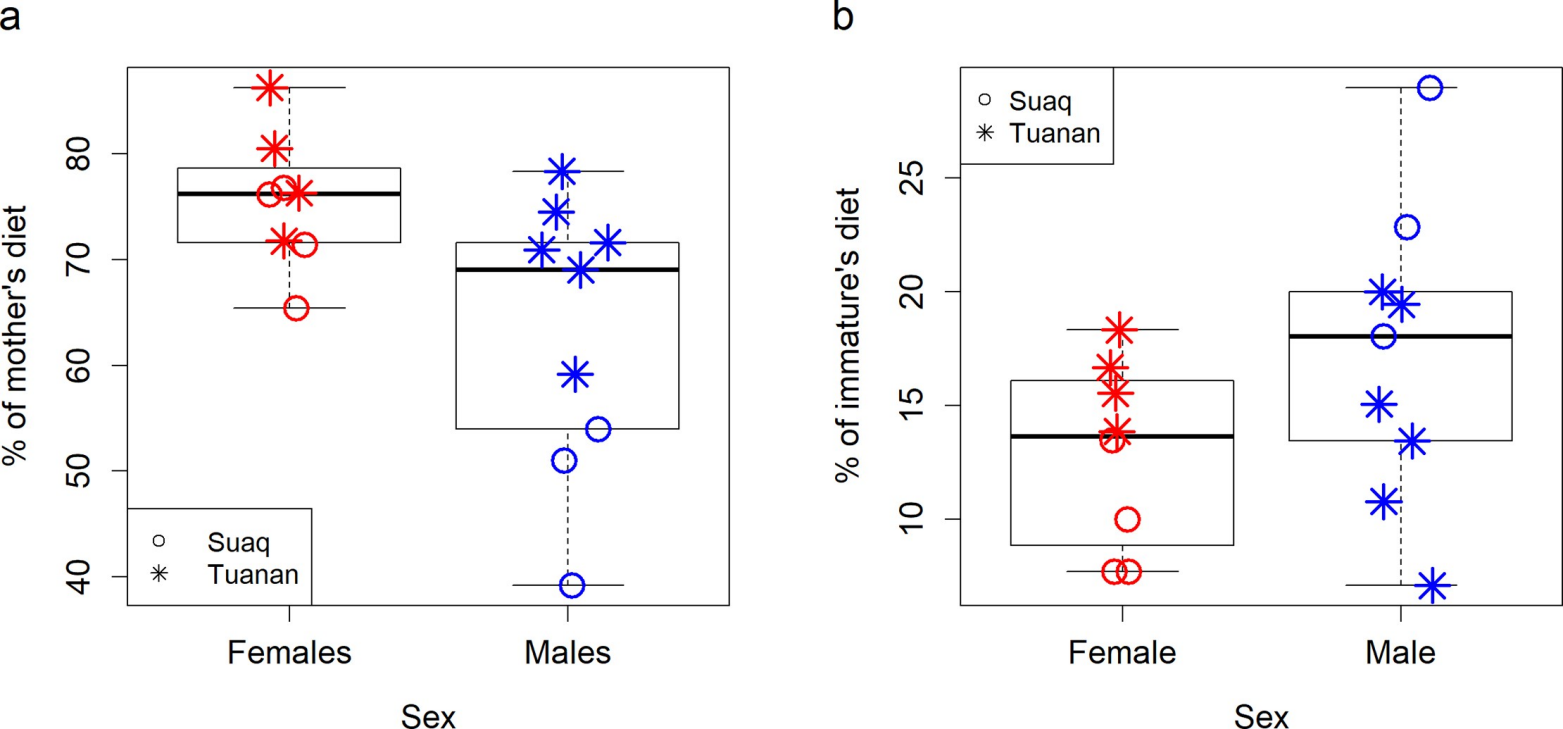
Diet repertoire similarity with the mother. (a) Percentage of the mother’s diet eaten by the offspring at the end of the dependency period (8 years) for immature males and females at Suaq and Tuanan with *N* = 17 (8 immature females and 9 immature males) computed dietary overlaps, based on a total of 670,748 feeding events of the immatures and their mothers collected during simultaneous focal follows. (b) Percentage of the diet of immature males and females which was not eaten by their mothers until the end of their dependency period (8 years) at Suaq and Tuanan with *N* = 17 (8 immature females and 9 immature males) computed dietary overlaps, based on a total of 670,748 feeding events of the immatures and their mothers collected during simultaneous focal follows. The underlying data for this figure can be found in S1 Data.

By the age of 8 years, the diets of immature females showed a significantly higher overlap of food items with their mothers’ diets compared to the diets of immature males (i.e., they ate a larger proportion of their mother’s food items; GLM_Gaussian_: P_SexMale_ = 0.004, Estimate_SexMale_ = −15.030, Std.Error_SexMale_ = 4.391; P_SiteTuanan_ = 0.010, Estimate_SiteTuanan_ = 14.570, Std.Error_SiteTuanan_ = 4.826; P_Nr.FeedingEvents_ = 0.953, Estimate_Nr.FeedingEvents_ < 0.001, Std.Error_Nr.FeedingEvents_ < 0.001, *N* = 17 computed dietary overlaps, based on 670,748 feeding events of the immatures and their mothers; Fig 3A).

Furthermore, by the age of 8 years, the diets of immature males comprised a higher percentage of food items which their mothers did not eat (GLM_Gaussian_: P_SexMale_ = 0.034, Estimate_SexMale_ = 7.198, Std.Error_SexMale_ = 2.991; P_SiteTuanan_ = 0.180, Estimate_SiteTuanan_ = -4.653, Std.Error_SiteTuanan_ = 3.287; P_Nr.FeedingEvents_ = 0.107, Estimate_Nr.FeedingEvents_ < 0.001, Std.Error_Nr.FeedingEvents_ < 0.001, *N* = 17 computed dietary overlaps, based on 196,636 feeding events of the immatures; Fig 3B). Of the food items that immature males ate but their mothers did not, 36.5% (51% at Suaq and 24.5% at Tuanan) were not part of the diet of any local adult female.

### The observed sex biases are specific to the feeding context

**Fig 4:**
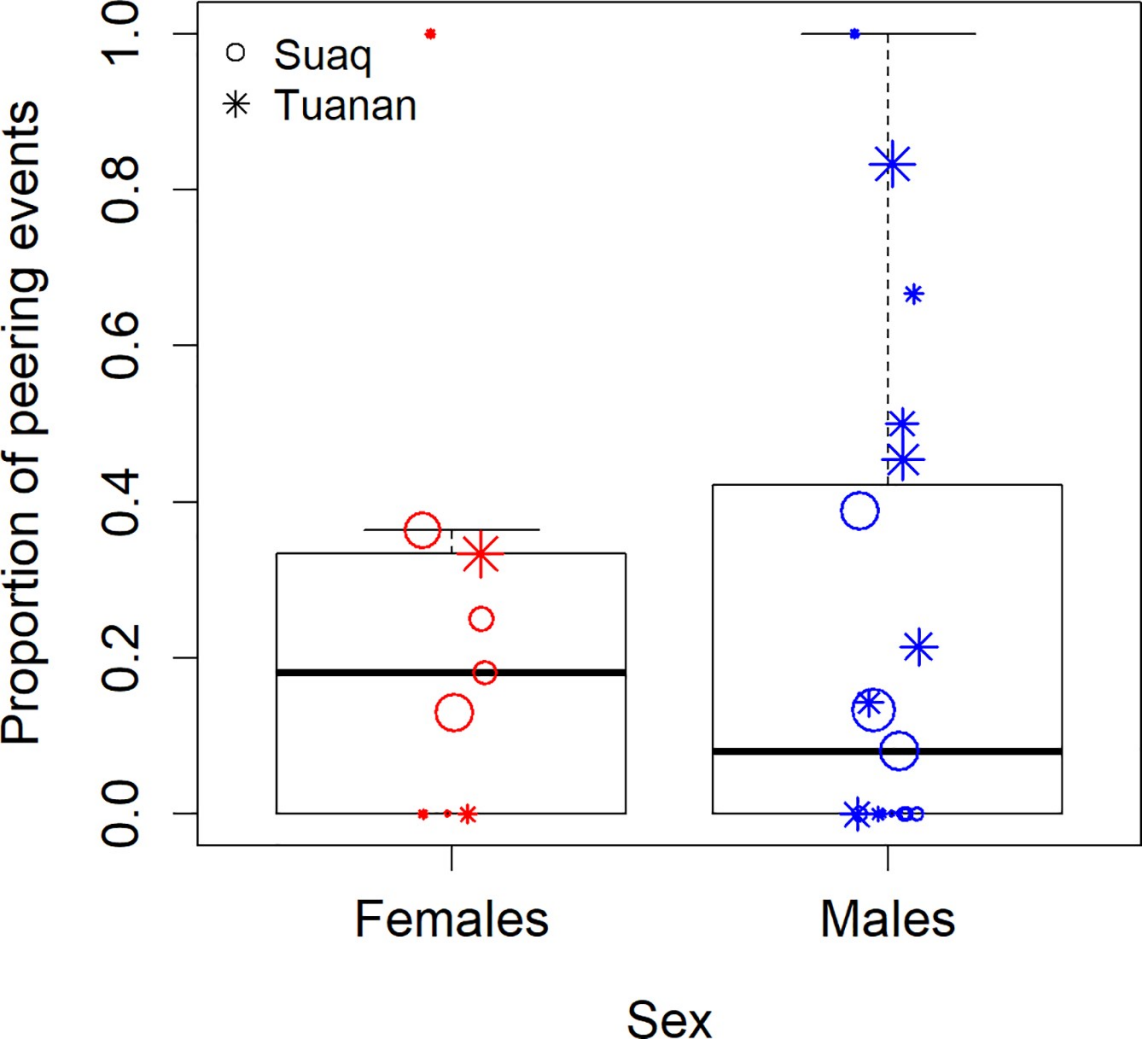
Non-subsistence peering: Proportion of all peering events outside of the feeding context that was directed at non-mother targets by immature females and males at Suaq and Tuanan. The symbol sizes correspond to the number of peering events per data point (*N* = 28 peering proportions based on a total of 479 peering event with 157 events by immature females and 322 events by immature males). The underlying data for this figure can be found in S1 Data.

In terms of peering at non-subsistence skills, we analyzed the proportion of peering events directed at non-mother individuals who were engaging in activities other than feeding, such as nesting (321 events, 67%), social interactions (52 events, 11%), resting (33 events, 7%), locomotion (15 events, 3%), and others. Because these data are sparse, we pooled all peering events outside the feeding context for each individual across age. In this data set, we did not find evidence for sex differences in non-subsistence peering (GLM_Gaussian_: P_SexMale_ = 0.738; Estimate_SexMale_ = −0.040, Std.Error_SexMale_ = 0.118; P_SiteTuanan_ = 0.026, Estimate_SiteTuanan_ = 0.261, St.Error_SiteTuanan_ = 0.111, *N* = 28 peering proportions based on 479 peering events outside the feeding context; Fig 4).

## Discussion

Our peering and association results support our main prediction that immature female and male orangutans differ in the development of their social interest. Interestingly, overall peering rates did not differ between the sexes, suggesting that immatures of both sexes have an equal amount of social interest in their association partners in general. Moreover, overall association rates (S2 Fig) as well as association rates with different age sex classes (S1 Table) did not differ between the different sexes. All in all, these patterns suggest that immature male and female orangutans have equal opportunities to learn from non-mother individuals during the age range focused on in our study (2 to 8 years). However, our results on the peering allocations and close associations showed that immature males and females are likely making different use of these opportunities.

Almost all immature females have an attentional preference for their mothers in the feeding context throughout their dependency period, such that they direct most peering events at her (Fig 1A). Peering at individuals other than the mother peaks during mid-infancy and decreases again, as does the proportion of association time immature females spend in close proximity to these individuals (Fig 1A). In contrast, over the course of the dependency period, immature males allocate an increasing share of their peering events in the feeding context to individuals other than the mother (Fig 1B). The association data, however, did not show an increase in the proportion of association time spent in close proximity of these individuals (Fig 1D). In combination, these results concerning the immature males suggest that, as they get older, immature males peer at non-mother individuals more frequently than predicted by their association patterns. This supports our prediction that immature males have an attentional preference for unfamiliar individuals.

Adult orangutans show limited levels of tolerance, and at times aggression toward older juvenile and adult conspecifics, in particular unrelated individuals [56,63]. This social intolerance may limit the proportion of association time that both sexes of immatures spend in close proximity to non-mother individuals as they get older. Our results suggest that this intolerance toward unrelated individuals seems to be particularly pronounced toward maturing females (Fig 1C), potentially because they will eventually settle in their natal area and thus become competition for the local resident females [57].

These patterns of development of attentional preferences in the feeding context are in line with our prediction that immature orangutans attend to individuals with the most relevant ecological knowledge. This can be explained by an attentional preference both toward sex-specific ecological skills and knowledge of same sex adults, and/or toward individuals with the most relevant ecological skills and knowledge based on their dispersal background. Unfortunately, because adult males are all immigrants, teasing these 2 options apart is difficult.

In line with our prediction, immature males were significantly more likely than immature females to attend to immigrant individuals (Fig 2B). Together with the increasing amount of immature males’ social attention being directed at individuals other than the mother (Fig 1B), these results align with the prediction of the second phase of social learning: With increasing age, the pool of targets for social attention widens [18]. Diversifying their knowledge spectrum and being able to learn from unfamiliar individuals may help males to cope with the challenges of dispersal. In contrast, upon reaching adulthood, female orangutans settle close to their mother’s home range. Therefore, to maximize their foraging efficiency, detailed knowledge on the local food resources is crucial for females. We found only a weak trend that immature males were more likely than immature females to peer at adult males, even though visualizing the data suggests that there is a difference (Fig 2A). However, notably, the sample size of this analysis of peering at adult males versus adult females (*N* = 198 peering events) was smaller than that of peering at resident versus immigrant individuals (*N* = 249 peering events), wherein we did see significant differences.

Sex-specific preferences to attend to feeding skills of same-sex adults are likely connected to sex differences in adult foraging profiles. Adult female orangutans and flanged males differ in aspects of their diets and foraging patterns, including the time they spend feeding, the breadths of their diets, their overall energy intake, and their nutrient intake profiles [49,51,52]. However, most of the peering events directed at adult males were directed at unflanged males (Fig 2C), who do not differ from females in most of these aspects apart from a higher protein intake [48]. Furthermore, there are no items that are exclusively eaten by males or females and can thus only be learned by attending to one sex [49,52].

As opposed to a specific preference to attend to individuals who have the most relevant ecological knowledge, a more general bias to observe adults of one’s own sex may be driving immatures’ sex differences in attentional biases. A general sex-specific attentional preference may serve to learn about sex-specific adult behavioral elements, as has been found in other primate species [34]. These general preferences should be apparent across different peering contexts and, arguably, be most pronounced in social contexts. However, we found no evidence for sex differences in peering at non-subsistence skills (Fig 4). This suggests that the sex differences that we did find in this study are caused by biases of immature males and females to specifically attend to the feeding skills and knowledge of particular association partners. However, the data set of these non-subsistence peering events (*N* = 479) was too small to allow us to control for the effects of age. This therefore limits the conclusions we can draw from these results.

Peering patterns at independent juveniles may help to further differentiate between the mechanisms underlying immatures’ sex-specific attention biases. Independent juveniles show less adult-specific behavior (such as behaviors in the sexual or mothering context). However, the sex-specific attention biases of the immatures were also apparent in peering at independent juveniles (Fig 2C). In line with our prediction concerning the effect of the residence status of the targets of social attention, the independent juveniles that were peered at by immature females were exclusively local residents (16 peering events by 4 immature females directed at 2 male and 1 female independent juveniles). Furthermore, immigrant independent juvenile peering targets (2 females and 1 male) were exclusively peered at by male immatures (20 peering events by 3 individuals). Although we currently lack sufficiently large sample sizes for conclusive testing of preferences for independent juveniles as peering targets, these results are suggestive that immature orangutans’ peering preferences are influenced by a target’s immigration status, more so that just its sex.

During the dependency period, an immature orangutan is highly dependent on its mother and actively maintains close proximity to her [60,64]. Therefore, the sex differences we see in the proportion of association time that immatures spent in close proximity to non-mother individuals might well be the result of mothers of female and male offspring having differing association patterns, as has been observed in chimpanzees [64]. To pin down whether or not differences in immatures’ social interest are indeed an expression of their own attentional preference or simply the result of differing association patterns of their mothers, we compared the proportion of association time that the immatures and their mothers spent in close proximity to their association partners. Overall, we found no difference in the proximity patterns of mothers of female and mothers of male offspring (S2 Table). The proximity patterns of the mothers of immature females did not follow the patterns of their offspring (S3A Fig, S2 Table). However, the proximity patterns of mothers with male offspring resembled those of their offspring (S3B Fig, S2 Table). Accordingly, when we included the mothers’ proximity patterns as a factor in the immatures’ allocation of social interest, model selection showed that the proportion of association time that mothers of immature males spend in close proximity to their association partners had a significant effect on the proportion of association time immature males spend in close proximity to these association partners (S3 Table). At the same time, the share of time spent in close proximity to their mothers develops similarly in immatures of both sexes (S3C and S3D Fig, S2 Table). In combination, these additional results suggest that the differing peering patterns shown by the immatures of either sex are not a result of mothers engaging in different association patterns depending on the sex of their offspring, but rather these differing peering patterns seem to reflect differing attentional preferences of the immatures. However, within associations, immature males appear to follow their mothers fine-scale proximity patterns or vice versa.

As a potential outcome of the differing attentional preferences, we found that by the time they stop being in constant association with their mothers, immature females have diets which are more similar to their mothers’ diets than immature males (Fig 3A). This result is in line with our prediction that immature females acquire more local knowledge than immature males. It is also consistent with what has been found in chimpanzees, where female offspring acquire termite fishing techniques that resemble the ones of their mothers more than males do [38]. We also found that immature males include a larger share of food items in their diets which their mothers do not eat (Fig 3B). Notably, several of the food items eaten by the immature males but not their mothers were not part of the diet of any of the local adult females (but were eaten by immigrants of both sexes). However, the extent to which males acquire their diets from social learning from non-mother individuals during the actual dispersal event and the extent to which they retain these diets in later life remains to be investigated, ideally by following these males into adulthood.

In this study, we did not address immatures’ learning from their semi-dependent older siblings (we excluded them from the analyses; see Methods), even though they provide potentially important learning opportunities for immature orangutans. Among chimpanzees, immatures learn from individuals that are the same age or older, including their older siblings [11], who seem to play an important role for the social development of immature chimpanzees in general [66,67]. In our peering data, the 5 immatures who overlapped with their older sibling in their constant association with their mothers during the age range analyzed here (i.e., 2 to 8 years), directed 0% to 7% (mean of 2%) of their peering events at their older sibling. All in all, these quantitative results suggest that learning from a semi-dependent older sibling is an opportunity which is not available to equal extents for all immature orangutans but may be used if available.

In conclusion, our results suggest that immature orangutans have attentional biases which prepare them for their sex-specific adult foraging niches: They pay attention to the individuals who are most likely to demonstrate ecological skills and knowledge that are most relevant to them. These learning biases appear to be specific to the feeding context and reflected in differences in the ecological knowledge acquired by immatures of the 2 sexes. Furthermore, these biases may be the result of increased social interest in feeding behavior of same sexed adult conspecifics and of individuals with corresponding dispersal ecologies. All in all, these results support the importance of sex-specific social learning for immature orangutans and most likely other primates.

## Methods

### Data collection

The data for this study were collected from 2007 to 2020 on a population of wild Sumatran orangutans (*Pongo abelii*) at Suaq Balimbing, South Aceh, Indonesia and from and 2003 to 2018 on a population of wild Bornean orangutans (*Pongo pygmaeus wurmbii*) at Tuanan, Central Kalimantan, Indonesia. Data were collected during focal animal follows, wherein the activity and associations of the focal individual were assessed through scans at 2-minute intervals.

Peering was defined as directly looking at the action of another individual sustained for at least 5 seconds and at a close enough range to enable the peering individual to observe the relevant details of the action (within 0 to 5 meters). The peering individual had to be facing the target individual and show signs of following the actions performed by the target individual (e.g., by head movements). We focused on immatures between 2 years and 8 years old because this age class shows the highest peering rates [68]. Before the age of 2 years, immature orangutans are mostly carried by their mothers and cannot approach association partners on their own [64]. Eight years marks the approximate end of the constant association of immatures with their mothers and thus the end of dependency on the mother, although this varies between individuals and sites [69]. After the age of 8, peering rates decrease significantly [6] and, because locally born males start to leave the research area at around 10 years old, sample sizes of older independent juveniles are female biased.

The peering data were collected during focal follows conducted by 64 experienced observers who recorded peering events of focal individual at 2-minute intervals (scan data set), as well as all peering events of any individual in sight between the scans (all occurrence data set). At both sites, interobserver reliability between experienced and new incoming observers was assessed via simultaneous follows of the same focal animal. Every observer whose data was included in the data sets of this study had reached a Cohen’s kappa [70] of k ≥ 0.8 with an experienced observer. For each peering event, the identity of the peerer, the identity of the peering target, and details about the context were noted. The total, unrestricted ad libitum data set of peering by immatures (0 to 8 years) comprised 2,543 peering events by 18 immatures (6 females and 12 males) at Suaq and 567 peering events of 17 immatures (4 females and 13 males) at Tuanan. The difference in sample size in the peering data between the 2 study sites resulted from a difference in overall peering rates between the populations (see [70]). Peering in the feeding context was defined as peering at an individual that was searching for, processing, or ingesting food.

To calculate overall hourly peering rates, we used the scan data set (i.e., only peering events that were recorded on the 2-minute intervals of the focal follows). We calculated a peering rate for each immature individual for each year, and we only included data points which comprised at least 25 follow hours. To assess the development of peering preferences, we used the all-occurrence peering data set to look at the proportion of all peering events directed at the mother of the focal animal versus at other association partners. We calculated peering proportions for each individual per year and only included data points that comprised a minimum of 5 peering events (range = 5 to 231, mean = 42.6) per individual per year. To analyze detailed non-mother peering target selection, we used all non-mother peering events for each immature individual and compared the proportion of peering targeted at (i) adult males (i.e., unflanged males and flanged males) versus adult females; and (ii) residents (adult females and resident independent juveniles) versus immigrants (unflanged males, flanged males, and nonresident independent juveniles, with independent juveniles being defined as independently ranging, but not yet adult sized and not yet sexually active, individuals). Whereas we used all data points in these statistical analyses, to visualize those patterns, we computed peering proportions at each class of peering target based on a minimum total of 5 peering events (range = 5 to 80, mean = 21.5) per individual.

To calculate overall hourly association rates and proportions of association time spent in close proximity to particular classes of conspecifics, we used the association compositions and interindividual distances that were recorded on the 2-minute intervals of the focal follows. Orangutans were defined as being in association when they were within 50 meters of each other and in close proximity when they were within 2 meters of each other. The association data from Suaq contained a total of 4,149 association hours of 18 immatures (5 females and 13 males) and 3,163 association hours of 13 mothers, and from Tuanan, 11,538 association hours of 26 immatures (8 females and 18 males) and 4,443 association hours of 16 mothers. Overall association rates and proportions of association time spent in close proximity were calculated for each individual for each year. For association rates, we only included data points that comprised a minimum of 25 follow hours (range = 25.5 to 345.8, mean = 139.5) per individual. For the proportion of association time spent in close proximity to non-mother association partners, we only included data points that were based on a minimum of 10 association hours (range = 21.6 to 171.0, mean = 47.8) with non-mother, adult, and independent juvenile association partners.

To calculate dietary overlap between immatures and their mothers, we compared the repertoires of all food items recorded as eaten by each immature and its mother from when an immature was born until it was 8 years old. These data were collected during simultaneous focal follows of the immatures and their mothers and comprised a total of 670,748 scans at which the focal individuals were feeding (196,636 scans of the immatures and 474,112 of their mothers). A single food item was defined as the combination of the species and the part of the species that was eaten. For plant parts, we differentiated between bark, flowers, fruits, leaves, pith, and other vegetative items, and for insect parts, we differentiated between the insect itself and its different products (i.e., honey or eggs). Additional food items included bird eggs and the meat of small mammals. Each food item requires a distinct sequence of processing steps before ingestion [72]. For each food item in the mother’s diet, we checked whether or not it was eaten by the offspring, and vice versa. Since repertoire overlaps may depend on the follow effort, we only included data that were collected during simultaneous follows of the immatures and their mothers and that comprised at least 1,000 feeding scans (range = 2,821 to 43,211, mean = 10,489) of the offspring (collected at 2-minute intervals, when the immature was between 0 and 8 years old). We also included follow effort as a factor in our analyses (see below).

To make our immature focal animals more comparable to one another, we excluded their semi-dependent older siblings from the non-mother peering target category for all analyses. A semi-dependent older sibling ranges in close association with its mother-offspring pair and shows high interaction rates with the dependent offspring, i.e., its younger sibling [71]. The presence of a semi-dependent older sibling, however, depends on birth order and the length of the mother’s interbirth interval. A total of 20 of the 31 individuals in our peering data set had no, or very limited, opportunities to peer at an older sibling because they were either firstborns or born after the older sibling had left their mothers. Only 11 individuals were exposed to an older sibling for an extended period of time (i.e., they were born when their older sibling was still ranging with their mother). Only 5 individuals were still exposed to an older sibling at the age of 2 years or older, i.e., during the time they were included in our data set.

### Ethics statement

As a strictly observational study on wild animals, we did not interact with our study animals in any way. All our research protocols were approved by the Ministry of Research, Technology and Higher Education (RISTEKDIKTI; Research Permit No.: 152/SIP/ FRP/SM/V/2012 and following) and adhered to the legal requirements of Indonesia.

### Statistical analyses

All statistical analyses and graphs were performed/made using the R programming Language [71]. To compare overall peering and association rates of the 2 sexes, we used generalized linear mixed models (GLMM, as implemented in the lme4 package in R [73]) with a Gaussian family distribution, using a full model approach. Aside from the main factor sex, we (here and throughout) included the factors site and age into the model because of their effects on peering and association rates found in previous studies [68]. We assessed the *P* values of the factors with the *cftest* function implemented in the *multcomp* package in R [74].

To examine sex-specific age effects on peering allocations and proportion of time immatures spent in close proximity, we used GLMMs with a Gaussian family distribution. A visual assessment of the data showed that these age effects were not linear and differed for the 2 sexes. Therefore, we fitted the models for female and male data separately. We used forward model selection to find the best fitting age effect by including age as a linear, quadratic, and linear and quadratic effect (see S4 Table for more detailed information about the model fits), using likelihood ratio test [75]. As a last step of this forward model selection process, we included site. The *P* values of the model selection process are summarized in S4 Table. The *P* values of the resulting final GLMMs were assessed with the *cftest* function implemented in the *multcomp* package in R [74].

To assess the effects of the immatures’ sex on their choices of peering targets (i.e., comparing the likelihood of peering at adult females versus adult males and resident versus immigrant individuals), we used GLMMs with a binomial family distribution, using a full model approach and including the factors sex, site, and age. For the analysis on peering at residents versus immigrants, the interaction between age and sex significantly improved the model fit, which is why we included it in the model.

In all GLMMs, we included the ID of the individual as a random factor, to account for the fact that individuals occur multiple times in the data set. Throughout all the abovementioned models, age was standardized through a Z-transformation.

To compare immature females’ and males’ dietary overlap with their mothers at the end of the dependency period (at the age of 8 years), we used generalized linear models (GLMs) with a Gaussian family distribution. Since dietary overlap is likely to be dependent on follow effort, we controlled for differences in follow effort by including the total number of 2-minute scans during which the focal was feeding as a factor in the model. To investigate sex differences in peering outside the feeding context, we used a GLM with a Gaussian family distribution and analyzed the proportion of total peering events that each immature individual directed at non-mother individuals engaging in activities other than feeding.

All model fits were examined visually to assess whether they satisfied model assumptions and to check for the presence of influential observations [76]. We assessed the stability of all our mixed models on the level of the random effects by excluding individuals one at a time. We found that the directions of these effects were consistent in all of the supported mixed models. The maximum of the variance inflation factors (VIFs, computed with the *vif* function in the *car* package in R [77]) of our independent variables across all models was 1.176, suggesting that our independent variables were not correlated with each other. For the binomial models, we tested for overdispersion and zero inflation using the *testDispersion* and *testZeroInflation* function in the *DHARMa* package in R [78]. The dispersion parameters of our models ranged from 0.9863 to 0.9996 and the ratio of observed to predicted zeros from 1.0016 to 1.0033.

## Supporting information

S1 Fig Overall peering rates. Average peering rates (peering events per hour) for immature males and females at Suaq and Tuanan (each data point is based on data collected on one individual within one age year, *N* = 63 peering rates based on 1,094 peering events in the feeding context by the immatures: 361 events by immature females and 733 events by immature males). The size of the symbols corresponds to the number of observation hours per peering rate (range = 33–277, mean = 108 hours). The underlying data for this figure can be found in S1 Data.
(TIF)

S2 Fig Overall association rates. Average association rates (cumulative association time per hour) for immature males and females at Suaq and Tuanan (each data point is based on data collected on one individual within one age year, *N* = 118 association rates based on 16,598 observation hours on the immatures: 5,486 hours on immature females and 11,112 hours on immature males). The size of the symbols corresponds to the number of observation hours per association rate (range = 25.5 to 345.8, mean = 139.5 hours). The underlying data for this figure can be found in S1 Data.
(TIF)

S3 Fig Associations of the mothers. Proportion of association time mothers of immature females (a) and males (b) spent in close proximity of their association partner (other than their dependent offspring) with *N* = 93 association proportions based on 7,524 association hours of the mothers. Proportion of association time immature females (c) and males (d) spent in close proximity of their mother for immatures at Suaq and Tuanan with *N* = 114 association proportions based on 15,649 association hours of the immatures with their mothers (5,060 hours on immature females and 10,589 hours on immature males). The lines depict the best model fit for each sample. Symbol sizes correspond to the number of association hours per data point. The underlying data for this figure can be found in S1 Data.
(TIF)

S1 Table Effects on associations with different classes of individuals. The effects of age, sex, and site on immatures’ associations with different classes of individuals, assessed using GLMMs with a Gaussian family distribution. Significant *P* values at the 5% criterion are bolded. GLMM, generalized linear mixed model.
(PDF)

S2 Table Model summaries for the association data of the mothers. Results of the model selections for (a) the effects of sex and age of the offspring and site on the proportion of association time mothers spent in close proximity of other association partners with *N* = 93 association proportions based on 7,524 association hours of the mothers; (b) the effects of age of the offspring and site on the proportion of association time the mothers of immature females and males spent in close proximity of other association partners with *N* = 93 association proportions based on 7,524 association hours of the mothers; and (c) the effects of age and site on the proportion of association time immature females and males spent in close proximity of their mothers with *N* = 114 association proportions based on 15,649 association hours of the immatures with their mothers (5,060 hours on immature females and 10,589 hours on immature males); analyzed with GLMMs with a Gaussian family distribution. Significant *P* values at the 5% criterion are bolded. GLMM, generalized linear mixed model.
(PDF)

S3 Table Effects of the mothers’ fine-scale proximity patterns on immatures’ attentional biases. Model comparisons of the minimal model to the model with the best fitting age term as well as of the model with the best fitting age term to the model including the proportion of association time the mother spent in close proximity of other individuals for (a) peering proportions directed at individuals other than the mother for immature females and males and (b) proportion of association time immature females and males spent in close proximity of individuals other than the mother. The best fitting models are indicated with bold font.
(PDF)

S4 Table Model selection via likelihood ratio tests. *P* values of the model comparisons between the minimal model (−) and the models with the different age components, between the models favored over the minimal model, and between the model with the best fitting age component and the model that additionally includes site for (a) peering proportions directed at individuals other than the mother for immature females and males and (b) proportion of association time immature females and males spent in close proximity of individuals other than the mother. The best fitting models are indicated with bold font.
(PDF)

S1 Data The Excel workbook contains 12 spreadsheets, each corresponding to a different figure or part of a figure. (XLSX)

## Abbreviations

GLM: generalized linear model
GLMM: generalized linear mixed model
VIF: variance inflation factor
