# Supplementary figures and images for: Immature wild orangutans acquire relevant ecological knowledge through sex-specific attentional biases during social learning
Source: PLoS Biol. 2021 May 19;19(5):e3001173. doi: 10.1371/journal.pbio.3001173 (PMC8133475; doi:10.1371/journal.pbio.3001173)

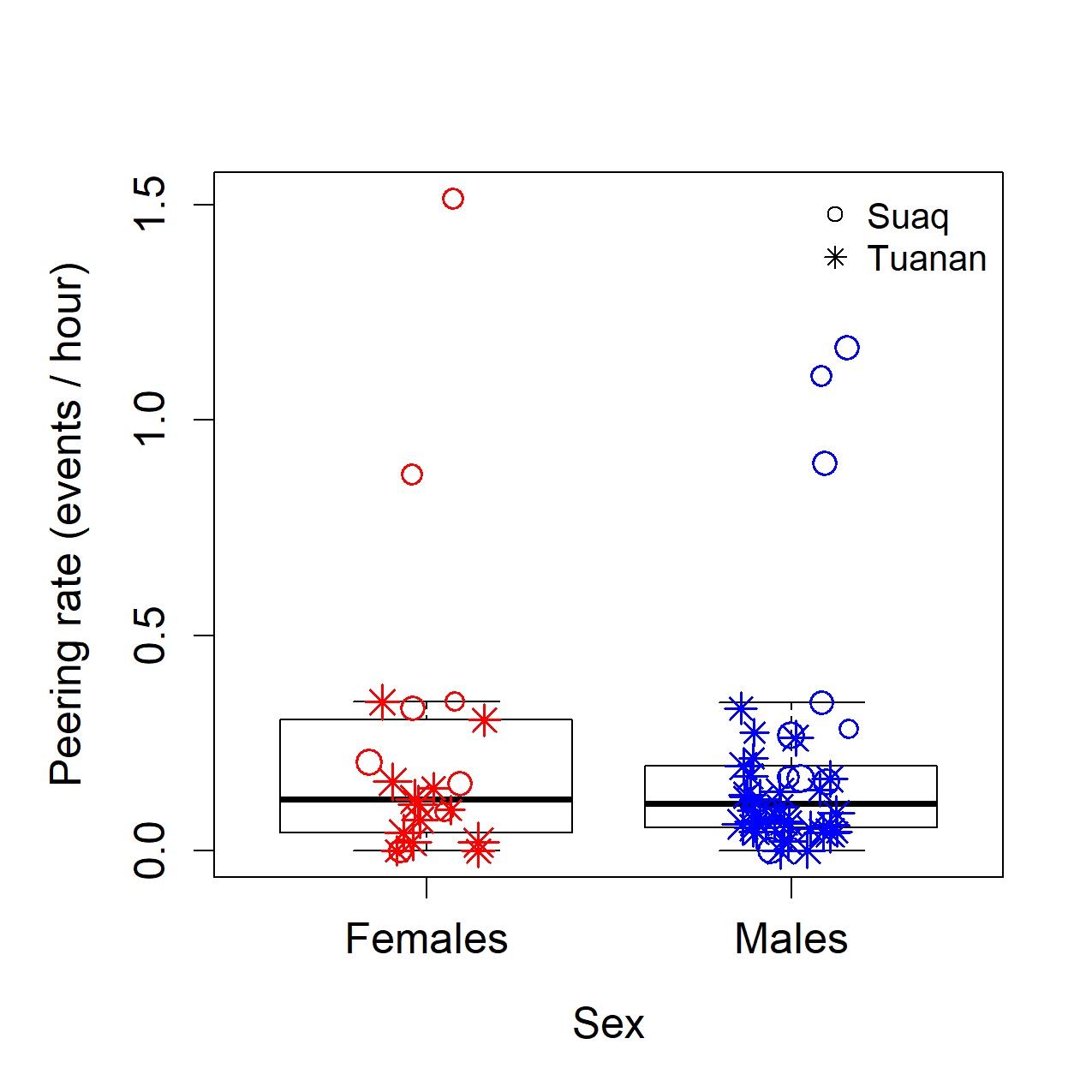

Supplement: S1 Fig — Average peering rates (peering events per hour) for immature males and females at Suaq and Tuanan (each data point is based on data collected on one individual within one age year, N = 63 peering rates based on 1,094 peering events in the feeding context by the immatures: 361 events by immature females and 733 events by immature males). The size of the symbols corresponds to the number of observation hours per peering rate (range = 33–277, mean = 108 hours). The underlying data for this figure can be found in S1 Data. (TIF) [file pbio.3001173.s001.tif]

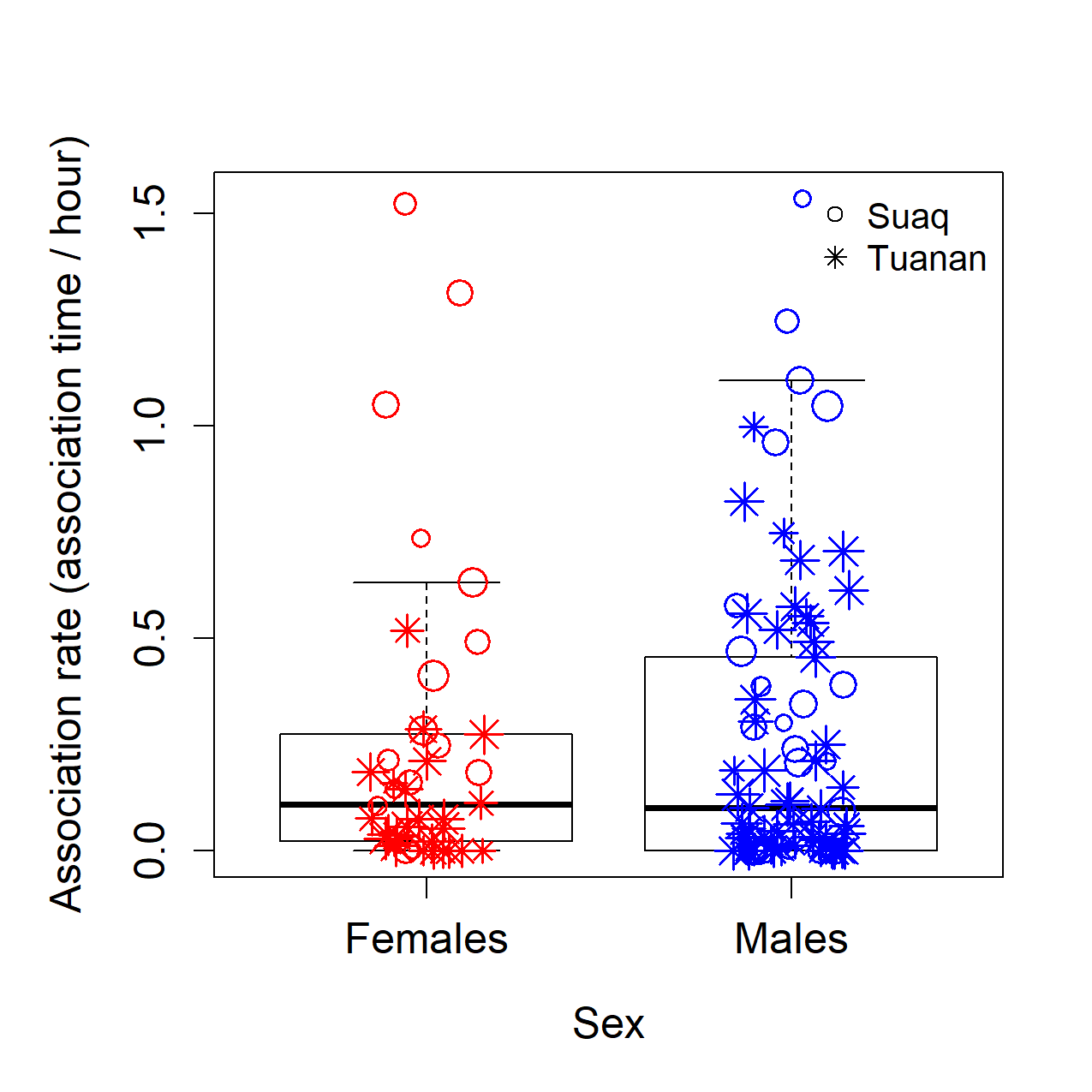

Supplement: S2 Fig — Average association rates (cumulative association time per hour) for immature males and females at Suaq and Tuanan (each data point is based on data collected on one individual within one age year, N = 118 association rates based on 16,598 observation hours on the immatures: 5,486 hours on immature females and 11,112 hours on immature males). The size of the symbols corresponds to the number of observation hours per association rate (range = 25.5 to 345.8, mean = 139.5 hours). The underlying data for this figure can be found in S1 Data. (TIF) [file pbio.3001173.s002.tif]

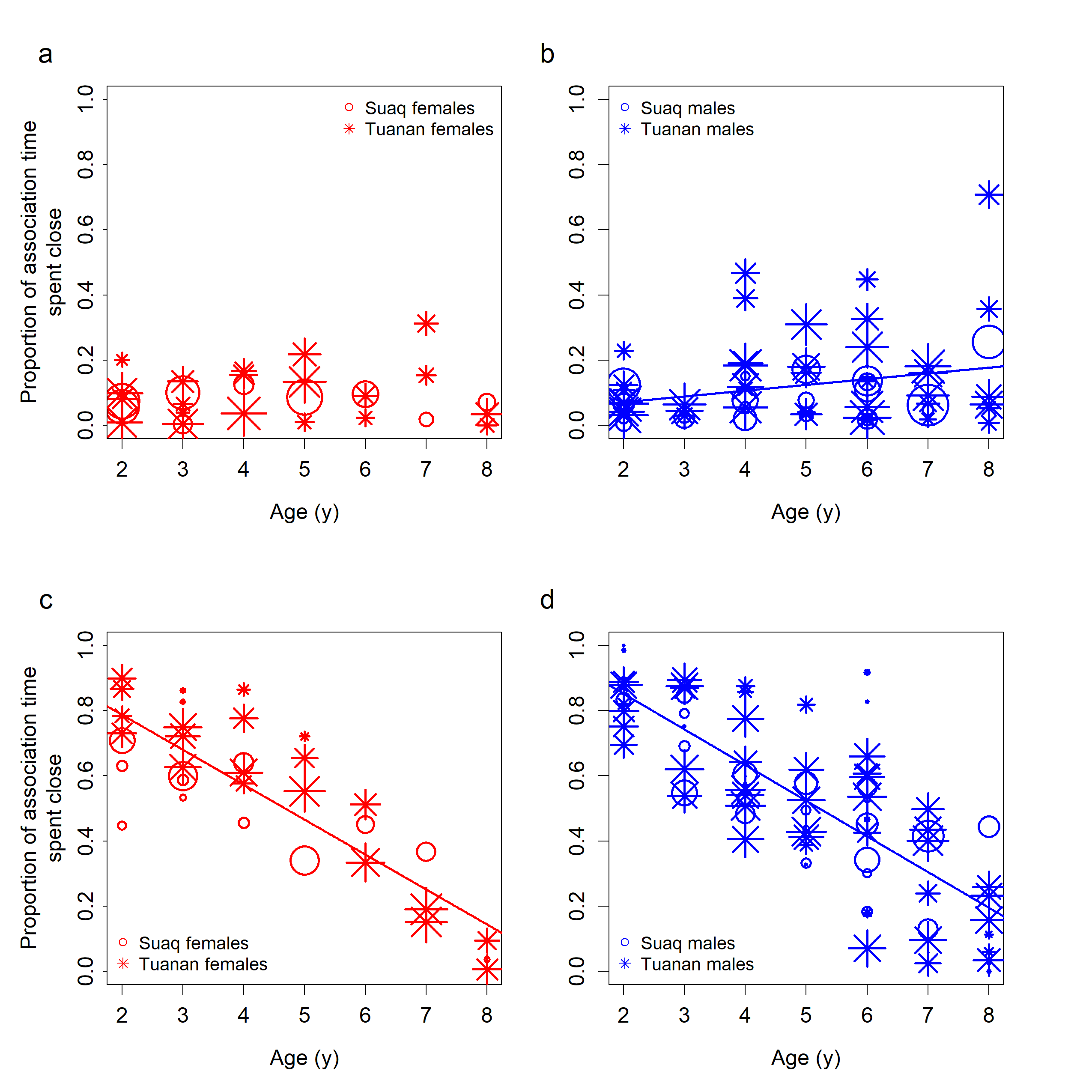

Supplement: S3 Fig — Proportion of association time mothers of immature females (a) and males (b) spent in close proximity of their association partner (other than their dependent offspring) with N = 93 association proportions based on 7,524 association hours of the mothers. Proportion of association time immature females (c) and males (d) spent in close proximity of their mother for immatures at Suaq and Tuanan with N = 114 association proportions based on 15,649 association hours of the immatures with their mothers (5,060 hours on immature females and 10,589 hours on immature males). The lines depict the best model fit for each sample. Symbol sizes correspond to the number of association hours per data point. The underlying data for this figure can be found in S1 Data. (TIF) [file pbio.3001173.s003.tif]
