# Supplementary material for: Immature wild orangutans acquire relevant ecological knowledge through sex-specific attentional biases during social learning
Source: PLoS Biol. 2021 May 19;19(5):e3001173. doi: 10.1371/journal.pbio.3001173 (PMC8133475; doi:10.1371/journal.pbio.3001173)
Supplement: S1 Table — The effects of age, sex, and site on immatures’ associations with different classes of individuals, assessed using GLMMs with a Gaussian family distribution. Significant P values at the 5% criterion are bolded. GLMM, generalized linear mixed model. (PDF) [file pbio.3001173.s004.pdf]

S1 Table. Effects on associations with different classes of individuals. The effects of age, sex, and site on immatures' associations with different classes of individuals, assessed using GLMMs with a Gaussian family distribution. Significant p-values at the 5% criteria are indicated with bold font.

Nr	Dependent variable	Effect	Effect type	Estimate	Std.Error	P-value
a)	Association rate of immatures with flanged males	Intercept	Intercept	0.029	0.015	0.059
		Age	Fixed	0.021	0.007	**0.005**
		Sex_{Male}	Fixed	0.017	0.015	0.259
		$Site_{Tuanan}$	Fixed	-0.018	0.015	0.232
		Individual	Random	-	-	-
a)	Association rate of immatures with unflanged males	Intercept	Intercept	0.075	0.036	**0.037**
		Age	Fixed	0.026	0.015	0.095
		Sex_{Male}	Fixed	0.049	0.036	0.185
		$Site_{Tuanan}$	Fixed	-0.069	0.036	0.057
		Individual	Random	-	-	-
c)	Association rate of immatures with adult females (excl. mother)	Intercept	Intercept	0.070	0.015	**<0.001**
		Age	Fixed	0.008	0.007	0.254
		Sex_{Male}	Fixed	-0.021	0.016	0.181
		$Site_{Tuanan}$	Fixed	-0.024	0.015	0.119
		Individual	Random	-	-	-
d)	Association rate of immatures with independent juveniles	Intercept	Intercept	0.029	0.034	0.399
		Age	Fixed	-0.022	0.014	0.122
		Sex_{Male}	Fixed	0.026	0.034	0.449
		$Site_{Tuanan}$	Fixed	0.001	0.034	0.984
		Individual	Random	-	-	-
