# Supplementary material for: Immature wild orangutans acquire relevant ecological knowledge through sex-specific attentional biases during social learning
Source: PLoS Biol. 2021 May 19;19(5):e3001173. doi: 10.1371/journal.pbio.3001173 (PMC8133475; doi:10.1371/journal.pbio.3001173)
Supplement: S2 Table — Results of the model selections for (a) the effects of sex and age of the offspring and site on the proportion of association time mothers spent in close proximity of other association partners with N = 93 association proportions based on 7,524 association hours of the mothers; (b) the effects of age of the offspring and site on the proportion of association time the mothers of immature females and males spent in close proximity of other association partners with N = 93 association proportions based on 7,524 association hours of the mothers; and (c) the effects of age and site on the proportion of association time immature females and males spent in close proximity of their mothers with N = 114 association proportions based on 15,649 association hours of the immatures with their mothers (5,060 hours on immature females and 10,589 hours on immature males); analyzed with GLMMs with a Gaussian family distribution. Significant P values at the 5% criterion are bolded. GLMM, generalized linear mixed model. (PDF) [file pbio.3001173.s005.pdf]

S2 Table. Model summaries for the association data of the mothers. Results of the model selections for a) the effects of sex and age of the offspring and site on the proportion of association time mothers spent in close proximity of other association partners with N = 93 association proportions based on 7’524 association hours of the mothers**;** b) the effects of age of the offspring and site on the proportion of association time the mothers of immature females and males spent in close proximity of other association partners with N = 93 association proportions based on 7’524 association hours of the mothers; and c) the effects of age and site on the proportion of association time immature females and males spent in close proximity of their mothers with N = 114 association proportions based on 15’649 association hours of the immatures with their mothers (5’060 hours on immature females and 10’589 hours on immature males); analyzed with GLMMs with a Gaussian family distribution. Significant p-values at the 5 % criteria are bolded.

Nr	Dependent variable	Sex	Effect	Effect type	Estimate	Std.Error	P-value
a)	Proportion of association time mothers spent in close proximity of other association partners	both	Intercept	Intercept	-0.018	0.043	0.673
			Offspring Sex_{Male}	Fixed	0.026	0.033	0.430
			$Age_{Offspring}$	Fixed	0.014	0.006	**0.015**
			$Site_{Tuanan}$	Fixed	0.068	0.032	**0.033**
			Individual	Random	-	-	-
b)	Proportion of association time mothers spent in close proximity of other association partners	Female Offspring	Intercept	Intercept	0.090	0.014	**<0.001**
			Individual	Random	-	-	-
		Male Offspring	Intercept	Intercept	0.119	0.021	**<0.001**
			$Age_{Offspring}$	Fixed	0.035	0.016	**0.031**
			Individual	Random	-	-	-
c)	Proportion of association time immatures spent in close proximity of their mothers	Female Offspring	Intercept	Intercept	0.526	0.048	**<0.001**
			Age	Fixed	-0.200	0.032	**<0.001**
			Individual	Random	-	-	-
		Male Offspring	Intercept	Intercept	0.555	0.026	**<0.001**
			Age	Fixed	-0.213	0.018	**<0.001**
			Individual	Random	-	-	-
