# Supplementary material for: Immature wild orangutans acquire relevant ecological knowledge through sex-specific attentional biases during social learning
Source: PLoS Biol. 2021 May 19;19(5):e3001173. doi: 10.1371/journal.pbio.3001173 (PMC8133475; doi:10.1371/journal.pbio.3001173)
Supplement: S3 Table — Model comparisons of the minimal model to the model with the best fitting age term as well as of the model with the best fitting age term to the model including the proportion of association time the mother spent in close proximity of other individuals for (a) peering proportions directed at individuals other than the mother for immature females and males and (b) proportion of association time immature females and males spent in close proximity of individuals other than the mother. The best fitting models are indicated with bold font. (PDF) [file pbio.3001173.s006.pdf]

S3 Table. Effects of the mothers' fine-scale proximity patterns on immatures' attentional biases. Model comparisons of the minimal model to the model with the best fitting age term as well as of the model with the best fitting age term to the model including the proportion of association time the mother spent in close proximity of other individuals for a) peering proportions directed at individuals other than the mother for immature females and males, and b) proportion of association time immature females and males spent in close proximity of individuals other than the mother. The best fitting models are indicated with bold font.

Nr	Dependent variable	Sex	Comparison	Addition	p-value
a)	Proportion of peering directed at non-mother individuals	Females	-	**Age^2**	**0.0160**
			Age^2	Proportion of association time mothers spent in close proximity of others	0.282
		Males	-	**Age + site**	**<0.001**
			Age + site	Proportion of association time mothers spent in close proximity of others	0.601
b)	Proportion of association time spent in close proximity individuals other than the mother	Females	-	**Age^2 + site**	**0.011**
			Age^2 + site	Proportion of association time mothers spent in close proximity of others	0.239
		Males	**-**	**Site**	**0.041**
			**Site**	Proportion of association time mothers spent in close proximity of others	**<0.001**
