# Supplementary material for: Immature wild orangutans acquire relevant ecological knowledge through sex-specific attentional biases during social learning
Source: PLoS Biol. 2021 May 19;19(5):e3001173. doi: 10.1371/journal.pbio.3001173 (PMC8133475; doi:10.1371/journal.pbio.3001173)
Supplement: S4 Table — P values of the model comparisons between the minimal model (−) and the models with the different age components, between the models favored over the minimal model, and between the model with the best fitting age component and the model that additionally includes site for (a) peering proportions directed at individuals other than the mother for immature females and males and (b) proportion of association time immature females and males spent in close proximity of individuals other than the mother. The best fitting models are indicated with bold font. (PDF) [file pbio.3001173.s007.pdf]

S4 Table. Model selection via likelihood ratio tests. P values of the model comparisons between the minimal model (-) and the models with the different age components, between the models favored over the minimal model, and between the model with the best fitting age component and the model that additionally includes site for a) peering proportions directed at individuals other than the mother for immature females and males, and b) proportion of association time immature females and males spent in close proximity of individuals other than the mother. The best fitting models are indicated with bold font.

Nr	Dependent variable	Sex	Comparison	Addition	p-value
a)	Proportion of peering directed at non-mother individuals	Females	-	Age	0.864
			-	**Age^2**	**0.016**
			-	**Age + Age^2**	**0.046**
			Age^2	Age + Age^2	0.568
			Age^2	Site	0.370
		Males	-	**Age**	**0.003**
			-	Age^2	0.584
			-	**Age + Age^2**	**0.012**
			Age	Age + Age^2	0.912
			Age	**Site**	**0.013**
b)	Proportion of association time spent in close proximity of individuals other than the mother	Females	-	Age	0.534
			-	**Age^2**	**0.037**
			-	Age + Age^2	0.106
			Age^2	**Site**	**0.030**
		Males	**-**	Age	0.6612
			**-**	Age^2	0.134
			**-**	Age + Age^2	0.264
			**-**	**Site**	**0.041**
